# Prebiotics enhance persistence of fermented-food associated bacteria in *in vitro* cultivated fecal microbial communities

**DOI:** 10.3389/fmicb.2022.908506

**Published:** 2022-09-02

**Authors:** Chloe M. Christensen, Car Reen Kok, Jennifer M. Auchtung, Robert Hutkins

**Affiliations:** Department of Food Science and Technology, Nebraska Food for Health Center, University of Nebraska–Lincoln, Lincoln, NE, United States

**Keywords:** fermentation, prebiotics, lactic acid bacteria, *Bifidobacterium*, GI microbiome, fermented foods

## Abstract

It is well established that the gastrointestinal (GI) microbiota plays a major role in human health. Dietary interventions, and consumption of fermented foods that contain live microbes, in particular, are among the approaches being investigated to modulate the GI microbiota and improve health. However, the persistence of fermented food-associated bacteria (FAB) within the GI tract is typically limited by host factors that limit colonization and competition with autochthonous microbes. In this research, we examined if the addition of prebiotics, dietary substrates that are selectively metabolized by microbes to improve health, would enhance the persistence of FAB. We evaluated the persistence of bacteria from three live microbe-containing fermented foods—kefir, sausage, and sauerkraut—in fecal microbial communities from four healthy adults. Fecal communities were propagated *in vitro* and were inoculated with fermented food-associated microbes from kefir, sausage, or sauerkraut at ~10^7^ CFU/mL. Communities were diluted 1:100 every 24 h into fresh gut simulation medium to simulate microbial community turnover in the GI tract. We measured the persistence of Lactobacillaceae from fermented foods by quantitative PCR (qPCR) and the persistence of other FAB through 16S rRNA gene sequencing. FAB were unable to persist *in vitro*, reaching undetectable levels within 96 h. Addition of prebiotics, including xylooligosaccharides and a mixture of fructooligosaccharides and galactooligosaccharides enhanced the persistence of some species of FAB, but the level of persistence varied by fecal donor, fermented food, and prebiotic tested. Addition of prebiotics also increased the relative abundance of *Bifidobacterium* species, which most likely originated from the fecal microbiota. Collectively, our results support previous *in vivo* studies demonstrating the transient nature of FAB in the GI tract and indicate that consumption of prebiotics may enhance their persistence.

## Introduction

It is now well established that the composition of the gastrointestinal (GI) microbiota has a profound influence on host–microbe interactions and the overall health status of humans and other animals (Manor et al., 2020). An altered or dysbiotic microbiota may also contribute to many contemporary diseases, including inflammatory bowel disease, obesity, and type 2 diabetes (Round and Mazmanian, 2009; Carding et al., 2015; Fan and Pedersen, 2021). Therefore, there is considerable interest in how diet and specific dietary compounds can modulate the GI microbiota and potentially redress a dysbiotic state (Wolter et al., 2021). One such approach is through consumption of probiotics, live microbes that confer a health benefit on the host (Hill et al., 2014), or prebiotics, substrates that are selectively utilized by host microorganisms conferring a health benefit (Gibson et al., 2017). Alternatively, consumption of fermented foods that contain live microbes has also been suggested to improve gut and systemic health (De Filippis et al., 2020; Marco et al., 2021; Wastyk et al., 2021).

Although a global dietary staple for thousands of years (Tamang et al., 2020), fermented foods have become especially popular recently, in part, because of their suggested nutritional properties (Marco et al., 2017; Leech et al., 2020). In addition to vitamins, minerals, proteins, and other macronutrients, the presence of live microbes in many fermented foods may also provide GI and systemic health benefits (Marco et al., 2021). Live microbes present in fermented foods are generally dominated by lactic acid bacteria (LAB), which includes members of the family Lactobacillaceae and members of the genera *Lactococcus* and *Streptococcus*; a few notable fungi and yeasts are also present (Wolfe et al., 2014). Ingested live microbes are subject to considerable barriers to growth and colonization during transit through the GI tract, including gastric acidity, bile salts, proteolytic and other digestive enzymes, antimicrobial peptides, and competition from autochthonous microbes (Kim et al., 2017; Han et al., 2021). Nonetheless, numerous studies have shown convincingly that LAB and other fermented food-associated bacteria (FAB), are able to reach and become transiently established in the human GI tract (Derrien and van Hylckama Vlieg, 2015; Park et al., 2015; Rossi et al., 2016; Milani et al., 2018; Pasolli et al., 2020; Wastyk et al., 2021).

The persistence of this so-called transient microbiome (De Filippis et al., 2020) is influenced by microbial traits, as well as the composition of the host microbiota, which differs significantly between individuals (Derrien and van Hylckama Vlieg, 2015). For example, in individuals who consumed an animal-based diet that included cheese and sausage, FAB were detectable during consumption of these fermented foods, but were absent after only 2 days of wash out (David et al., 2014). In contrast, several studies have demonstrated that persistence of transient microbes is enhanced if they are able to utilize dietary carbohydrates (David et al., 2014; Maldonado-Gómez et al., 2016). Thus, prebiotics, which are utilized by many LAB (Gibson et al., 2017), may provide a basis for enhancing persistence of FAB in addition to other health promoting benefits.

In this study, we used a previously described *in vitro* human fecal microbiota model (Kok et al., 2019) to assess the persistence of FAB in communities of fecal microbes. This model uses a batch cultivation platform with daily dilution into fresh gastrointestinal simulation medium (GSM) under strict anaerobic conditions to simulate turnover in the distal colon. This model provides a basis for distinguishing between bacteria that persist from bacteria that wash out or are displaced. We investigated whether FAB from kefir, sausage, or sauerkraut persisted over time following inoculation into communities of human fecal microbes. In parallel, we tested whether the addition of prebiotics to these fecal communities would enhance persistence of FAB through provision of specialized resources. Our studies focused primarily on the persistence of members of the Lactobacillaceae family, as this family includes many of the lactic acid bacteria commonly associated with fermented foods (Zheng et al., 2020), although we also monitored the persistence of other FAB through 16S rRNA gene sequencing. We found that FAB were unable to persist over time in fecal communities. However, the addition of prebiotics enhanced the persistence of Lactobacillaceae, at least transiently, and also supported the persistence of *Bifidobacterium* species that were most likely autochthonous. These results, which are consistent with those from human feeding studies, indicate that fermented foods should be consumed regularly to maintain populations of FAB in the gut and that consumption of prebiotics may enhance the maintenance of fermentation-associated Lactobacillaceae.

## Materials and methods

### Fecal sample collection

Fecal samples were collected from five healthy adult volunteers as per approved Institutional Review Board (IRB) protocols (IRB 20160616139). Participants were 19 years or older, had no known gastrointestinal diseases, were not regular consumers of yogurt, and had not consumed antibiotics or probiotic supplements in the previous 6 months. Enrolled participants were provided with a commode specimen collection kit (Thermo Fisher Scientific, Waltham, MA, United States), and samples were collected as previously described (Kok et al., 2019). Briefly, freshly collected samples were returned to the lab in sealed containers and transferred to an anaerobic chamber (Bactron IV anaerobic chamber; Sheldon Manufacturing, Cornelius, OR, USA) with a gas atmosphere of 5% H_2_, 5% CO_2_ and 90% N_2_. Samples were diluted 1:10 (final concentration = 100 mg/mL) in anaerobic phosphate-buffered saline (PBS) and homogenized using a handheld immersion blender (Hamilton Beach, Glen Allen, VA, United States). Multiple aliquots (2 mL) of processed fecal slurries were stored at −80°C until use.

To minimize potential interference with autochthonous Lactobacillaceae, fecal samples were screened by quantitative PCR (qPCR) using primers specific to the 16S rRNA gene from Lactobacillaceae (see below) to determine baseline levels. Of the five samples tested (Fecal 1–5), one sample (Fecal 5) contained Lactobacillaceae above the threshold of detection (~4 log CFU/mL, equivalent to 10^5^ Lactobacillaceae/g of feces) and therefore was not used in subsequent experiments. While exclusion of fecal samples that contained detectable levels of Lactobacillaceae may have introduced bias against fecal communities that could support the growth of Lactobacillaceae, it was necessary to include this exclusion criteria to facilitate tracking of fermented food-associated Lactobacillaceae.

### Media used in this study

Fecal and sausage homogenates were prepared in PBS. PBS contained (per liter): sodium chloride (8.0 g), potassium chloride (0.2 g), sodium phosphate dibasic (1.44 g), and potassium phosphate monobasic (0.24 g). PBS was adjusted to pH 7 and sterilized by autoclaving. *In vitro* fecal cultures were performed in gut simulation medium (GSM) as previously described (Kok et al., 2019). GSM contained (per liter): peptone (2 g), yeast extract (2 g), bile salts (0.5 g), sodium bicarbonate (2 g), sodium chloride (0.1 g), potassium phosphate dibasic (0.08 g), magnesium sulfate heptahydrate (0.01 g), calcium chloride hexahydrate (0.01 g), L-cysteine hydrochloride (0.5 g), Tween 80 (2 mL), and 0.025% (w/v) resazurin solution (4 mL), and was sterilized by autoclaving. Sterile solutions of vitamin K_1_ (10 μL) and hemin (1 mL of 5 mg/mL dissolved in dimethylsulfoxide) were added after autoclaving. Medium was pre-reduced in the anaerobic chamber for 48 h prior to use and anaerobic conditions were confirmed based on the redox state of resazurin. To test the effects of prebiotics, xylooligosaccharides (XOS; Prenexus Health, Gilbert, AZ, United States), fructooligosaccharides (FOS; Beneo, Parsippany, NJ, USA), or galactooligosaccharides (GOS, FrieslandCampina, LE Amersfoort, Netherlands) were added to GSM. XOS, FOS, and GOS (>95% purity) were dissolved in distilled water, filter sterilized and added to GSM. XOS was used alone and added at a final concentration of 1% w/v. Because of limited availability of pure GOS, it was combined with FOS as a 1:1 mixture (0.5% w/v of each compound). Similar FOS:GOS mixtures have been widely used previously (Vos et al., 2006; van Hoffen et al., 2009; Zhang et al., 2021). de Man, Rogosa, and Sharpe (MRS, Difco-Beckton Dickinson, Franklin Lakes, NJ, United States) and Elliker agars (Difco-Beckton Dickinson) were used for enumeration of lactic acid bacteria (LAB) from fermented foods. *Lactobacillus* Selection (LBS) Agar, prepared as described previously (Rogosa et al., 1951) from reagents obtained from various manufacturers, was used to isolate potential LAB from fecal samples. MRS broth was used to enrich for LAB from sauerkraut samples (described below) and to test growth of isolates (described below). GSM, GSM with added prebiotics (1% XOS or 0.5% FOS,0.5% GOS), or GSM + 1% glucose (prepared in distilled water and filter sterilized before addition to sterile GSM) were also used to measure growth of LAB isolated from fermented foods and fecal samples.

### Fermented foods used in this study

Fermented foods included one commercial brand of sauerkraut (Farmhouse Culture), two brands of kefir [Lifeway (Kefir A) and Green Valley (Kefir B)] and two types of dry fermented sausage [Gusto Napoli (Sausage A) and Gusto Chorizo (Sausage B)], all purchased from a local retail market in Lincoln, Nebraska, United States in 2019. All products were used within 7 days of purchase. According to the label declarations, these products were unheated and claimed to contain live fermentation microorganisms. Live LAB were enumerated from these foods by spread plating samples from serial dilutions (10^3^–10^9^) onto MRS and Elliker agar. Plates were incubated, both anaerobically and aerobically, at 32 and 37°C. This was done in duplicate. The median value and range of CFU/mL or CFU/g across all plating and incubation conditions are reported in Table 1. For sauerkraut and kefir, the liquid portions were used for LAB enumeration and *in vitro* fecal cultivation (see below). Sausage was diluted 1:100 in PBS (10 mg/mL final concentration) and homogenized using a stomacher (Stomacher 80 Biomaster, Seward, Bohemia, NY, United States) prior to LAB enumeration and *in vitro* fecal cultivation.

**Table 1:** Lactobacillaceae concentrations in fermented foods determined by selective plating onto MRS and Elliker agar and qPCR.

Product	Selective plating^*^	qPCR
*Kefir*		
Lifeway	3.5 × 10^8^ (2.0 × 10^8^–8.0 × 10^8^) CFU/mL	9.0 × 10^8^ CFU/mL
Green Valley	5.0 × 10^8^ (9.0 × 10^6^–4.0 × 10^9^) CFU/mL	2.0 × 10^8^ CFU/mL
*Sausage*		
Gusto Napoli	1.5 × 10^9^ (<1.0 × 10^4^–3.0 × 10^9^) CFU/g	2.0 × 10^9^ CFU/g
Gusto Chorizo	2.5 × 10^9^ (2.0 × 10^8^–2.0 × 10^10^) CFU/g	5.0 × 10^9^ CFU/g
*Sauerkraut*		
Farmhouse Culture	3.0 × 10^7^ (5.0 × 10^3^–4.0 × 10^8^) CFU/mL	3.0 × 10^7^ CFU/mL

* Median (Range) reported.

### *In vitro* fecal cultivation to test the persistence of fermented food-associated bacteria

The *in vitro* fecal cultivation protocol was described previously (Kok et al., 2019) and is intended to mimic natural gastrointestinal competition and flux through daily serial dilution (1:100) of batch cultures in GSM, a medium that simulates nutrient conditions in the distal colon. Fecal slurries, prepared as described above, were removed from frozen storage, homogenized by vortexing, and filtered through four layers of cheese cloth. Then, 3 mL of homogenized, filtered fecal slurry were added to 6 mL of anaerobic GSM in a 15 mL tube. This dilution resulted in a final concentration of 300 mg fecal sample/*in vitro* culture. The initial concentration of fecal microbes in *in vitro* cultures was estimated to be about ~3 × 10^9^ CFU/mL based upon previous studies from our lab with similarly prepared fecal samples that demonstrated total anaerobic plate counts of fecal microbes to be 10^10^ CFU/g (Davis et al., 2010). 1 mL of a fermented food (kefir, sauerkraut brine, or homogenized sausage slurry), prepared as described above, was added to each fecal culture. Fermented foods were pre-incubated in the anaerobic chamber for 30 min prior to addition to culture.

**Figure 1:**
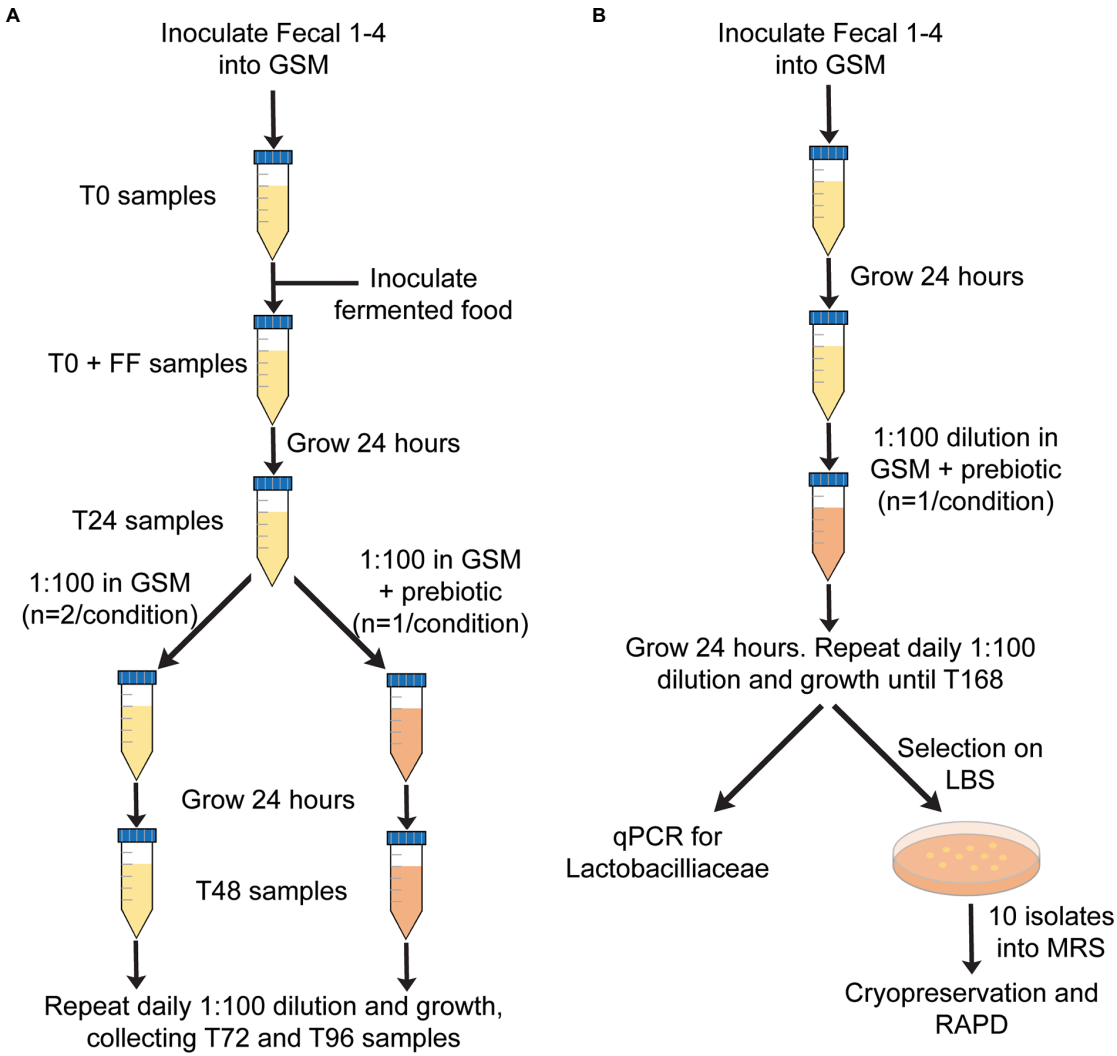
Overview of *in vitro* fecal culture experimental design. **(A)** Approach used to test the persistence of FAB over time (0–96 h) in the presence or absence of prebiotics. **(B)** Approach used to test whether prebiotics would enhance the growth of autochthonous Lactobacillaceae.

Samples were incubated at 37°C under strict anaerobic conditions (Bactron IV anaerobic chamber with a gas atmosphere of 5% H_2_, 5% CO_2_ and 90% N_2_) and diluted 1:100 every 24 h into fresh GSM for 96 h. Four different fecal samples (Fecal 1–4) were tested for each treatment. Each sample was treated as a single experimental unit. In the initial experiment, daily dilutions were performed into fresh GSM for 96 h (Figure 1A). In a second experiment to test the effects of prebiotics on the persistence of FAB, daily dilutions were performed in GSM, GSM + 1% XOS, or GSM + 0.5% FOS:0.5% GOS (Figure 1A). 1 mL samples were obtained from *in vitro* fecal cultures at baseline (T0), immediately after inoculation of fermented food (T0 + FF), and after 24, 48, 72, and 96 h (T24, T48, T72, T96). Samples were stored at −20°C and used later for DNA extraction, qPCR and 16S rRNA gene sequencing as described below.

### DNA extraction and quantification of Lactobacillaceae by qPCR

DNA from fermented foods and *in vitro* cultivated fecal communities was extracted using the phenol-chloroform method as described by Martínez et al. (2015). Briefly, 1.0 mL of sample was centrifuged at 8,000 × *g* for 5 min at room temperature and washed four times in ice-cold PBS. Recovered cells were resuspended in lysis buffer (200 mM sodium chloride, 10 mM Tris, 0.8 mM EDTA, pH =8) and transferred to sterile bead beating tubes containing 0.3 g of 0.1 mm zirconium beads (Research Products International, Mount Prospect, IL, United States). Samples were homogenized in a MiniBeadbeater (BioSpec, Bartlesville, OK, United States) for one two minute cycle. The cell lysate layer was extracted three times with an equal volume of phenol-chloroform-isoamyl alcohol (25:24:1) and twice with an equal volume of chloroform-isoamyl alcohol (24,1). DNA was precipitated from the aqueous layer with 2X volume ice cold ethanol. Precipitation was enhanced by incubation at −20°C for 30 min. DNA was pelleted by centrifugation at 20,000 × *g* for 20 min at 4°C. Ethanol was removed, samples were air-dried for 30 min, and DNA was resuspended in 100 μL of DNase-free water. DNA quantity and quality was determined with a NanoDrop-1,000 spectrophotometer (Thermo Fisher Scientific).

Quantification of Lactobacillaceae was performed by qPCR using a Mastercycler Realplex2 (Eppendorf AG, Hamburg, Germany) with *Lactobacillus* group primers described by Walter et al. (2001). In addition to *Lactobacillus*, these primers target other Lactobacillaceae species, such as *Pediococcus*, *Leuconostoc* and *Weissella*. Each 25 μL reaction contained 12.5 μL of qPCR SYBR green (2X Maxima SYBR Green Master Mix; Thermo Fisher Scientific), 0.5 μL of 20 mM forward and reverse primers (F: 5′- AGC AGT AGG GAA TCT TCCA6–3′;R: 5′- ATT YCA CCG CTA CAC ATG-3′; Walter et al., 2001), 8.5 μL of ultrapure water, and 3 μL of DNA template (50 ng/μL). Reactions were performed in duplicate for each sample. qPCR cycling conditions were an initial denaturation at 95°C for 5 min, followed by 40 cycles of 95°C for 15 s, 58°C for 20 s, and 72°C for 30 s. A standard curve was generated using tenfold serial dilutions of DNA isolated from a pure culture of *Lactiplantibacillus plantarum* 299 V that had been enumerated by serial dilution and plating on MRS agar. *C_t_* values of standards were plotted against their log_10_ CFU/mL values and used to calculate CFU/mL concentrations for Lactobacillaceae in fermented foods and *in vitro* fecal microbial cultures. The limit of detection based on qPCR was equivalent to 10^4^ CFU/mL of *L. plantarum*.

### *In vitro* fecal cultivation to test the ability of prebiotics to enhance the growth of fecal LAB

Although we were unable to detect Lactobacillaceae in fecal samples 1–4 by qPCR, we hypothesized that incubation of fecal samples in the presence of prebiotics could enhance the growth of autochthonous Lactobacillaceae in fecal samples that could be present below the limit of detection. To test this hypothesis, each of the four fecal samples were cultured *in vitro* in GSM as described above. Twenty four hours after inoculation, cultures were diluted 1:100 in GSM with 1% XOS or a mixture of 0.5% FOS and 0.5% GOS. Dilutions were repeated every 24 h into the same media for 168 h (Figure 1B). 1 mL samples were removed after 168 h of incubation and used for enumeration of Lactobacillaceae by qPCR (described below) and by serial dilution in PBS (10^1^–10^7^) followed by spread plating 100 μL of undiluted and diluted samples on *Lactobacillus* Selection (LBS) Agar (Rogosa et al., 1951). LBS agar plates, spread in duplicate, were incubated anaerobically at 37°C for 48 h prior to counting.

### Characterization of potential Lactobacillaceae from fecal cultures and sauerkraut

To further characterize potential Lactobacillacae enumerated on LBS agar from *in vitro* fecal cultures after prebiotic enrichment, 10 colonies from each fecal culture from each prebiotic were selected from LBS agar after 48 h of anaerobic incubation at 37°C and transferred into fresh MRS broth (*n* = 20 cultures/fecal sample; 80 cultures total). Cultures were grown for 24 h prior to collection of two 1.0 mL samples for DNA extraction and cryopreservation of cultures in MRS with 30% v/v sterile, anaerobic glycerol and storage at −80°C.

We also isolated potential Lactobacillaceae from sauerkraut for comparison to fecal culture isolates. Isolates were obtained directly from sauerkraut brine and from sauerkraut brine exposed to prebiotics to enrich for Lactobacillaceae. For direct isolation, 100 μL of sauerkraut brine were spread on a single MRS agar plate and incubated at 37°C anaerobically for 48 h. For prebiotic enrichment, sauerkraut brine was diluted 1:10 (v/v) in MRS broth supplemented with either 1% XOS or 0.5% FOS: 0.5% GOS and incubated at 37°C for 24 h. 100 μL of enriched sample was spread onto a single MRS agar and incubated at 37°C anaerobically for 48 h. Ten colonies per sample were then selected and inoculated into fresh MRS broth (*n* = 30 cultures total). As above, cultures were grown for 24 h prior to collection of two 1.0 mL samples for DNA extraction and cryopreservation of cultures.

### Random amplified polymorphic DNA fingerprinting

A total of 110 isolates, 80 derived from fecal cultivation and 30 derived from sauerkraut as described above were examined by RAPD fingerprinting. Briefly, RAPD-PCR with primer M13V (5′-GAG GGT GGC GGT TCT-3′; Meroth et al., 2003) was performed in a 50 μL reaction mixture containing 250 pmol of primer M13V, 10 mM of each dNTP, 12 mM of reaction buffer, 1.2 U of Taq polymerase and 4 μL of DNA (50 ng/μL). PCR was performed with a Mastercycler Reaplex2 (Eppendorf AG) as described by Meroth et al. (2003). Briefly, reactions were held under the following cycling conditions, an initial denaturation at 94°C for 45 s, followed by three cycles of 94°C for 3 min, 40°C for 5 min, and 72°C for 5 min. Then, an additional 32 cycles of 94°C for 1 min, 60°C for 2 min, and 72°C for 3 min were carried out. PCR products were separated by electrophoresis in 1.5% agarose gels containing ethidium bromide at 40 V for 4.5 h. Gels were imaged under UV light illumination. A total of 27 isolates with unique RAPD profiles were selected for 16S rRNA gene sequencing. PCR with primers 8F (5′-AGA GTT TGA TCC TGG CTC AG-3′; Weisburg et al., 1991) and 1391R (5′-GAC GGG CGG TGT GTR CA-3′; Turner et al., 1999) was conducted as previously described (Kok et al., 2019). Briefly, each reaction of 50 μL volume contained 20 μM amount of each primer, 10 mM of each dNTP, 12 mM of reaction buffer, 1.2 U of Taq polymerase and 1 μL of DNA (50 ng/μL). Reactions were performed with a Mastercycler Reaplex2 (Eppendorf AG) with cycling conditions of an initial denaturation at 94°C for 3 min, followed by 32 cycles of 94°C for 1 min, 56°C for 45 s, and 72°C for 2 min, with an ending extension period at 72°C for 10 min. PCR products were purified using a QIAquick PCR purification kit (Qiagen, Hilden, Germany) and quantified using a NanoDrop ND-1000 spectrophotometer. Purified PCR products were sequenced by the Genomics Core Facility at Michigan State University using dideoxy chain termination sequencing on an Applied Biosystems 3730xl DNA Analyzer (Thermo Fisher Scientific). At least one isolate was selected from each fecal sample, fermented food, and their corresponding prebiotic treatments. Preliminary identification of isolates was conducted using NCBI BLASTn, with taxonomies assigned based on an identity threshold of ≥98.0% sequence similarity. Table 2 contains taxonomic information about sequenced isolates; Supplementary Table 1 also contains 16S rRNA gene sequences.

**Table 2:** Identity of potential Lactobacillaceae isolated from sauerkraut brine, prebiotic-enriched sauerkraut brine, and *in vitro* fecal cultures enriched with prebiotic.

Source	Prebiotic	Species	Identity (%)	GenBank accession
Sauerkraut	None	*Lactobacillus paracasei*	100	KF030743.1
Sauerkraut	None	*Lactobacillus paracasei*	100	ON795864.1
Sauerkraut	None	*Lactobacillus paracasei*	100	MT549175.1
Sauerkraut	None	*Lactobacillus paracasei*	99.86	ON631824.1
Sauerkraut	None	*Lactobacillus paracasei*	100	ON795864.1
Sauerkraut	XOS	*Lactobacillus buchneri*	99.86	MT045842.1
Sauerkraut	XOS	*Lactobacillus paracasei*	100	KF030743.1
Sauerkraut	XOS	*Pediococcus parvulus*	99.88	MK575520.1
Sauerkraut	XOS	*Lactobacillus paracasei*	99.11	MT549175.1
Sauerkraut	FOS:GOS	*Lactobacillus paracasei*	100	ON795864.1
Sauerkraut	FOS:GOS	*Lactobacillus paracasei*	100	ON795864.1
Sauerkraut	FOS:GOS	*Lactobacillus paracasei*	100	MT549175.1
Fecal Sample 1	XOS	*Enterococcus faecalis*	100	ON796012.1
Fecal Sample 1	XOS	*Enterococcus faecalis*	100	ON782146.1
Fecal Sample 2	XOS	*Bifidobacterium longum*	98.05	CP096771.1
Fecal Sample 2	XOS	*Bifidobacterium longum*	99.87	ON631733.1
Fecal Sample 3	XOS	*Bifidobacterium longum*	99.0	ON631733.1
Fecal Sample 3	XOS	*Bifidobacterium longum*	99.26	ON631733.1
Fecal Sample 3	XOS	*Enterococcus faecalis*	100	ON796012.1
Fecal Sample 4	XOS	*Enterococcus avium*	100	MT604783.1
Fecal Sample 4	XOS	*Bifidobacterium longum*	100	ON631733.1
Fecal Sample 4	XOS	*Enterococcus faecalis*	100	ON845622.1
Fecal Sample 1	FOS:GOS	*Enterococcus faecium*	100	MH819639.1
Fecal Sample 1	FOS:GOS	*Enterococcus faecium*	100	ON715739.1
Fecal Sample 2	FOS:GOS	*Enterococcus faecalis*	100	ON796012.1
Fecal Sample 3	FOS:GOS	*Enterococcus faecium*	100	MH819639.1
Fecal Sample 4	FOS:GOS	*Enterococcus durans*	100	ON564885.1

### Growth of individual Lactobacillaceae isolates in GSM with prebiotics

One Lactobacillaceae isolated from sauerkraut (*Lactobacillus paracasei* #1, Table 2), three Lactobacillaceae isolated from sauerkraut following XOS enrichment (*L. paracasei* #2, *Lactobacillus buchneri*, and *Pediococcus parvulus*, Table 2), and two species isolated from fecal sample 4 following XOS enrichment (*Bifidobacterium longum*, *Enterococcus faecalis,* Table 2) were grown from frozen stock on MRS agar at 37°C anaerobically for 48–72 h. Colonies were inoculated into GSM + 1% glucose and grown anaerobically overnight at 37°C. Cell density of overnight cultures was measured by optical density at 600 nm and used to adjust inocula to the same concentration across samples. Overnight cultures were diluted 1:10 v/v in fresh medium (GSM, GSM + 1% XOS, GSM + 0.5% FOS:0.5% GOS, or MRS as positive control) in duplicate and incubated anaerobically at 37°C for 24 h. Optical density at 620 nm (OD620) was measured every 20 min using a Sunrise plate reading spectrophotometer (Tecan, Männedorf, Switzerland) in an anaerobic chamber (Coy Type A unheated chamber, Coy Laboratory Products, Grass Lake, MI, United States) with a 5% H_2_, 5% CO_2_, 90% N_2_ atmosphere. Normalized OD620 values for each time point were calculated by subtracting the initial OD620 from the raw OD620 value for each sample. Maximum normalized OD620 for each sample was then identified using Microsoft Excel. The experiment was repeated in duplicate, and all four values (2 replicates from duplicate experiments) were plotted as geometric mean ± geometric standard deviation using GraphPad Prism 9.1.2. Potential statistical significance of differences between maximal OD620 values across media types for each isolate were determined using one-way ANOVA with Dunn’s correction for multiple testing on log transformed data. All comparisons with *p* < 0.05 were reported.

### 16S rRNA gene sequencing and analysis

A total of 51 samples were selected for 16S rRNA gene sequencing analysis. Samples were sequenced from each type of fermented food (*n* = 3 samples, which were sequenced in duplicate) and *in vitro* fecal cultures from all four fecal samples inoculated with each fermented food at 0 (*n* = 12 samples) and 96 h of cultivation in GSM, GSM + 1% XOS, or GSM + 0.5% FOS:0.5% GOS (*n* = 36 samples). All samples were collected from the second experiment that included growth in GSM and GSM + prebiotics described above. DNA was extracted as described above and DNA concentration was determined with Quant-iT DsDNA HS kit (Thermo Fisher Scientific) according to manufacturer’s protocol. The V4 region of the 16S rRNA gene (Caporaso et al., 2010) was amplified with Phusion polymerase using Illumina barcoded primers 515F and 806R as previously described (Auchtung et al., 2015). Briefly, reactions contained 4 μL of DNA template (at average concentrations of 11 ng DNA/ μL), 1X Phusion High Fidelity Buffer (Thermo Fisher Scientific), 200 μM dNTPs (Thermo Fisher Scientific), 10 nM primers, and 0.225 units of Phusion Polymerase (Thermo Fisher Scientific). Samples were amplified with the following cycle conditions: an initial denaturation at 98°C for 30 s, followed by 26 cycles of 98°C for 10 s, 51°C for 20 s and 72°C for 1 min, followed by final extension at 72°C for 1 min. Duplicate PCR reactions for each sample were pooled and samples were combined at equal concentrations prior to sequencing on an Illumina MiSeq using a 2 × 250 bp kit according to manufacturer’s protocol (Illumina, San Diego, CA, United States).

Fastq sequences were processed through mothur v 1.41.3 essentially as described (Kozich et al., 2013; Auchtung et al., 2015) with the modifications described below. Sequences were quality filtered then aligned to the V4 region of sequences in the SILVA database (release 132). Any potential chimeric sequences were identified by uchime and removed from further analysis. Sequences were classified with the Bayesian classifier using SILVA reference database (>80% confidence); any Archeal, Chloroplast, or Mitochondrial sequences were removed. Pairwise distance matrices were calculated and used to cluster sequences into Operational Taxonomic Units (OTUs) with ≥99% average nucleotide identity. A total of 1,854 OTUs were identified; taxonomy for each OTU was assigned using the majority consensus SILVA taxonomy for sequences within that OTU. A complete list of OTUs and their sequence abundances across samples can be found in Supplementary Table 2. Good’s coverage was calculated for each sample to ensure adequate sequence depth (0.997111–0.999887) and is reported in Supplementary Table 2. Sequences were randomly subsampled to 17,708 sequences per sample prior to further sequence analysis using ATIMA v3.1.2.^1^ A complete list of OTUs and abundances following subsampling can be found in Supplementary Table 3. Good’s coverage, while reduced (0.9944–0.9995), was still very high indicating sufficient sequencing coverage; this data is also reported in Supplementary Table 3. Shannon’s Diversity index, the total number of OTUs per sample, and the total number of reads per genus for each sample was determined using ATIMA. Alpha diversity measures were plotted using GraphPad Prism v 9.1.2, which was also used to determine statistically significant differences in these measures. For pairwise comparisons, a Student’s t-test with Brown and Forsythe correction for unequal variances was used. For three-way comparisons, a one-way ANOVA with Brown and Forsythe correction for unequal variances and Welch’s correction for multiple comparisons was used. To track persistence of fermented food bacteria, OTUs detected in duplicate fermented food samples were identified in Microsoft Excel and abundance across all samples was collated into Supplementary Tables 4–6. For visualization, OTUs from these tables were filtered to remove OTUs that were below 0.1% abundance (<17 reads) in duplicate samples of each fermented food. Percent relative abundances were visualized using GraphPad Prism. For identification of genera that differed significantly between samples treated with prebiotics, ANCOM-BC (Lin and Peddada, 2020) v 1.60 implemented in RStudio v2022.02 running R version 4.2.0 was used to identify genera with differential abundance in 96 h cultures that were grown in the presence of XOS, FOS:GOS, or without prebiotic. For visualization in GraphPad Prism, statistically significant taxa identified through ANCOM-BC were filtered in Microsoft Excel to identify those taxa present at ≥0.1% in at least 25% of samples in one of the treatment conditions compared. A complete list of ANCOM-BC results is reported in Supplementary Table 7.

## Results

### Lactobacillaceae from fermented foods do not persist in fecal communities cultured *in vitro*

**Figure 2:**
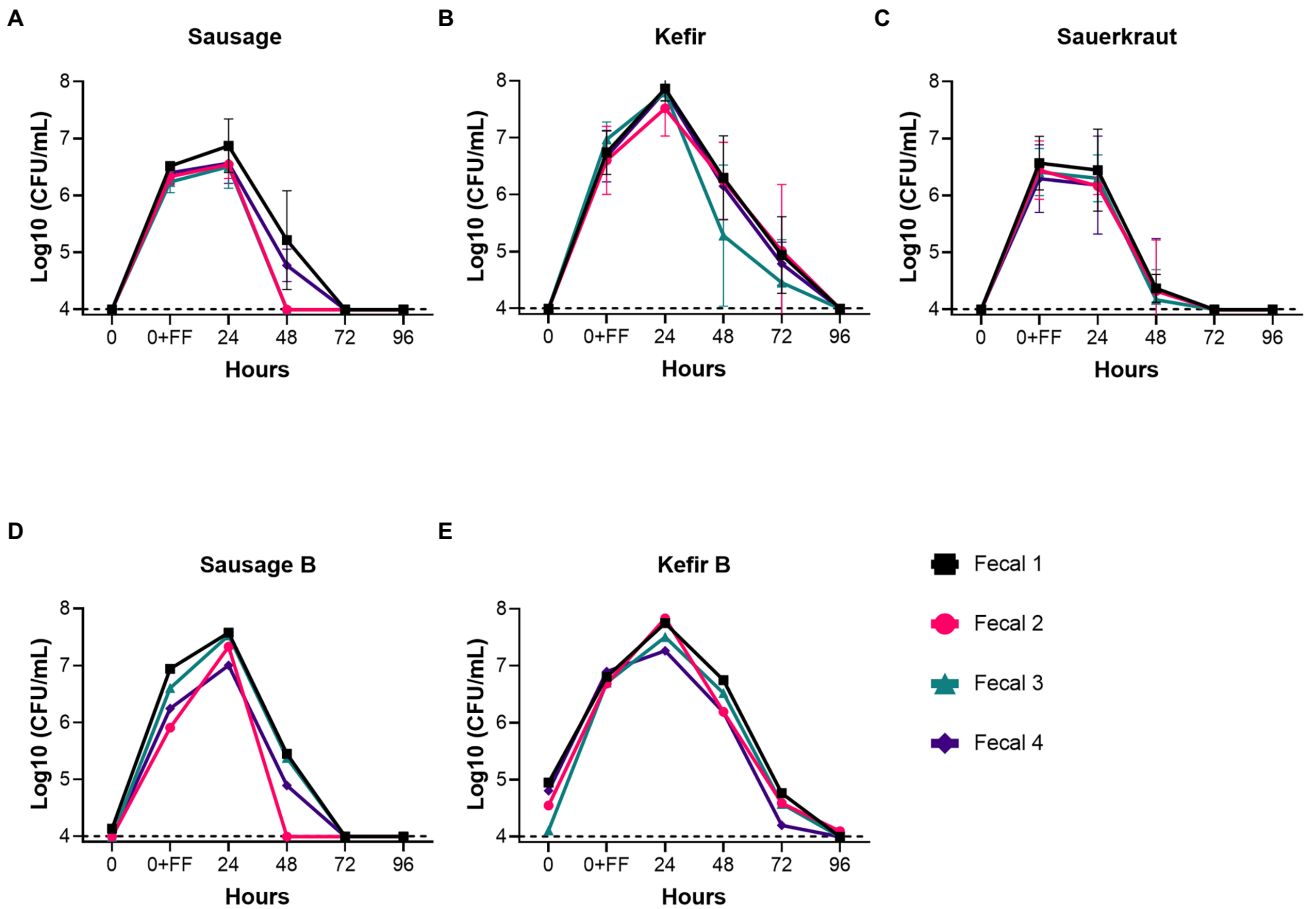
Fermented food-associated Lactobacillaceae were unable to persist under conditions simulating the distal GI tract. The persistence of Lactobacillaceae from two commercial brands of sausage **(A,D)**, two commercial brands of kefir **(B,E)**, or sauerkraut **(C)**, was monitored over time (0–96 h) by qPCR in simulated gastrointestinal environments colonized with microbes from one of four fecal samples. Samples were collected at the time points indicated. The dashed line designates the limits of detection (10^4^ CFU/mL). Each colored line indicates a unique fecal microbiota (Fecal 1, black squares; Fecal 2, pink circles; Fecal 3, teal triangles; Fecal 4, purple diamonds). Values for the graphs in **(A–C)** are the mean + SD of two biological replicates.

Fecal cultures from four healthy individuals (Fecal 1–4) were inoculated into *in vitro* culture vessels containing GSM. Based upon previous studies from our lab (Davis et al., 2010), the concentration of fecal bacteria following inoculation were estimated to be 3 × 10^9^ CFU/mL. One of three different fermented foods, sausage, kefir, or sauerkraut, was then introduced at a final concentration of 10% v/v (10^6^–10^7^ CFU/mL, Table 1). Cultures were then diluted 1:100 daily in fresh GSM for 4 days to simulate turnover in the GI tract as outlined (Figure 1A). As Lactobacillaceae are the predominant bacteria in the fermented foods we tested, we measured the persistence of Lactobacillaceae through qPCR as an initial proxy for all fermented food-associated bacteria. (qPCR values were converted to approximate CFU/mL values based upon standard curves with *L. plantarum*). Based on qPCR, Lactobacillaceae levels at inoculation ranged from 10^6^–10^7^ CFU/mL, which were consistent with cell counts and qPCR concentrations determined for the fermented foods (Table 1). These levels were maintained for the first 24 h of cultivation for sausage and sauerkraut (Figure 2); for kefir, an average increase of approximately one log was observed during this time. However, Lactobacillaceae levels fell below the limit of detection (10^4^ CFU/mL) between 48 and 96 h in all four donor cultures; similar results were observed in duplicate trials (Figures 2A–C). To confirm that these results were not due to the specific commercial brands that were tested, a second brand of kefir and sausage was also tested for persistence in *in vitro* cultures, with similar results observed (Figures 2D,E).

### Prebiotics enhance persistence of fermentation-associated Lactobacillaceae

**Figure 3:**
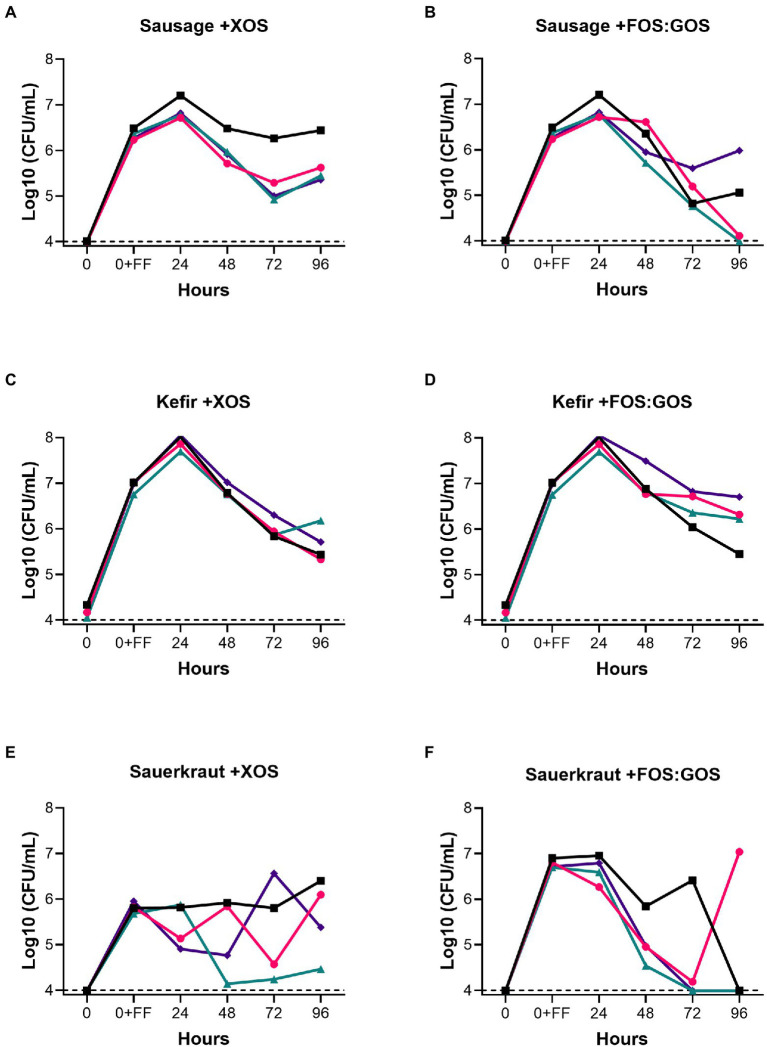
Prebiotics enhance the persistence of fermented food-associated Lactobacillaceae. The persistence of Lactobacillaceae from sausage **(A,B)**, kefir **(C,D)** or sauerkraut brine **(E,F)** were monitored over time (0–96 h) in simulated gastrointestinal environments colonized with one of four fecal samples in GSM supplemented with 1% XOS **(A,C,E)** or GSM supplemented with a mixture of 0.5% FOS: 0.5% GOS **(B,D,F)**. Samples were collected at the time points indicated. Baseline indicates the limits of detection (10^4^ CFU/mL). Each colored line indicates a unique fecal microbiota (Fecal 1, black squares; Fecal 2, pink circles; Fecal 3, teal triangles; Fecal 4, purple diamonds).

Because GSM simulates conditions of the distal colon, GSM does not contain fermentable carbohydrates that could support the growth of Lactobacillaceae from fermented foods. Therefore, we tested whether addition of 1% XOS or a mixture of 0.5% FOS and 0.5% GOS to *in vitro* fecal communities cultured in GSM would affect the persistence of Lactobacillaceae from sauerkraut, sausage, or kefir as outlined in Figure 1A. XOS, FOS, and GOS are prebiotics known to enhance the growth in Lactobacillaceae, as reported by Gibson et al. (2017). For Lactobacillaceae originating from sausage, addition of XOS promoted persistence for 96 h in all four donor samples at 10^5−^10^7^ CFU/mL (Figure 3A). In contrast, addition of the FOS:GOS mixture supported the persistence of Lactobacillaceae from sausage in only two communities (Fecal 1 and 4, 10^5^–10^6^ CFU/mL), while Lactobacillaceae levels declined to the limit of detection in the two remaining communities (Fecal 2 and 4, <10^4^ CFU/mL, Figure 3B). For Lactobacillaceae originating from kefir, both the XOS and the FOS:GOS mixture supported the persistence of Lactobacillaceae at 10^5^–10^7^ CFU/mL (Figures 3C,D). Finally, Lactobacillaceae originating from sauerkraut persisted when supplemented with XOS (10^5^–10^6^ CFU/mL for Fecal 1, 2 and 4, 10^4^ for Fecal 3; Figure 3E), but declined to the limit of detection in three of four cultures supplemented with the FOS:GOS mixture (Fecal 1, 3, 4; Figure 3F).

### Prebiotics do not enrich for autochthonous Lactobacillaceae in fecal communities

**Figure 4:**
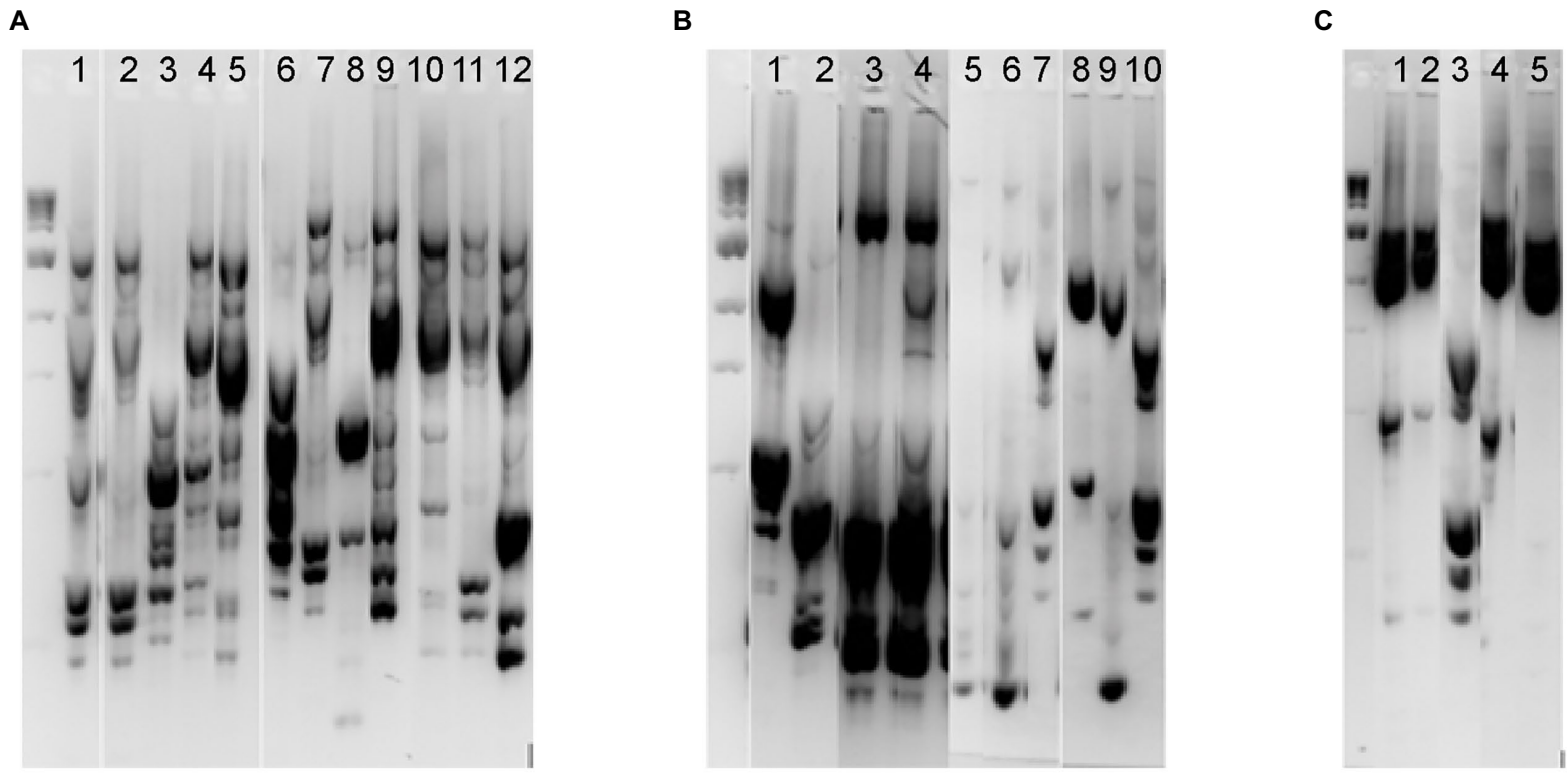
RAPD fingerprinting demonstrated differences in putative Lactobacillaceae in sauerkraut and prebiotic-enriched fecal communities. Unique RAPD fingerprints of the 27 bacterial isolates from *Lactobacillus* selection agar that were chosen for 16S rRNA gene sequencing. **(A)** Isolates from sauerkraut brine before (Lanes 1–5) and following enrichment with 1% XOS (Lanes 6–9) or 0.5% FOS and 0.5% GOS (Lanes 10–12). **(B)** Isolates from fecal samples enriched with XOS; samples were from Fecal 1 (Lanes 1–2), Fecal 2 (Lanes 3–4), Fecal 3 (Lanes 5–7), and Fecal 4 (Lanes 8–10). **(C)** Isolates from fecal samples enriched with 0.5% FOS and 0.5% GOS; samples were from Fecal 1 (Lanes 1–2), Fecal 2 (Lane 3), Fecal 3 (Lane 4), and Fecal 4 (Lane 5). The full gel images from which these unique fingerprints were cropped are shown in Supplementary Figure 1, which indicates those lanes that were selected for this figure.

While our initial screening indicated that Lactobacillaceae levels in fecal samples 1–4 were below the limit of detection, we hypothesized that *in vitro* cultivation of fecal communities in the presence of prebiotics could promote emergence of autochthonous Lactobacillaceae rather than promoting persistence of fermented food-associated Lactobacillaceae. To test this hypothesis, we cultivated fecal communities in GSM in the presence of 1% XOS or 0.5% FOS:0.5% GOS with daily dilution for 168 h as outlined (Figure 1B). After 168 h, we measured levels of Lactobacillaceae by qPCR (all samples were below the limit of detection) and enumerated (Table 3) and isolated *Lactobacillaceae* and related species by selective plating on LBS Agar. Twenty representative colonies (ten colonies/prebiotic) were isolated from each fecal sample (80 colonies total). In addition, 30 colonies were isolated from sauerkraut: 10 colonies were isolated directly from sauerkraut brine and 10 colonies each were isolated from sauerkraut brine that had been incubated in the presence of each prebiotic for 24 h. Genomic fingerprints of these isolates were determined by RAPD, which resulted in 27 unique banding patterns (Figure 4; Supplementary Figure 1). Sequencing of the 16S rRNA gene verified that these were 27 unique isolates (Table 2; Supplementary Table 1). We found that Lactobacillaceae could only be isolated from sauerkraut and prebiotic-enriched sauerkraut samples. Fecal communities cultured *in vitro* in the presence of prebiotics yielded no Lactobacillaceae; isolates instead were classified as members of the Bifidobacteriaceae or Enterococcaceae families (Table 2).

**Table 3:** Levels of potential Lactobacillaceae after *in vitro* fecal culture in the presence of prebiotic determined by selective plating on LBS agar.

Prebiotic substrate	Sample	Concentration
*XOS*		
	Fecal Sample 1	5 × 10^3^ CFU/mL
	Fecal Sample 2	2 × 10^4^ CFU/mL
	Fecal Sample 3	3 × 10^3^ CFU/mL
	Fecal Sample 4	9 × 10^3^ CFU/mL
*FOS:GOS*		
	Fecal Sample 1	8 × 10^2^ CFU/mL
	Fecal Sample 2	4 × 10^3^ CFU/mL
	Fecal Sample 3	1 × 10^3^ CFU/mL
	Fecal Sample 4	6 × 10^3^ CFU/mL

### Fermented food-associated Lactobacillaceae grow in GSM

**Figure 5:**
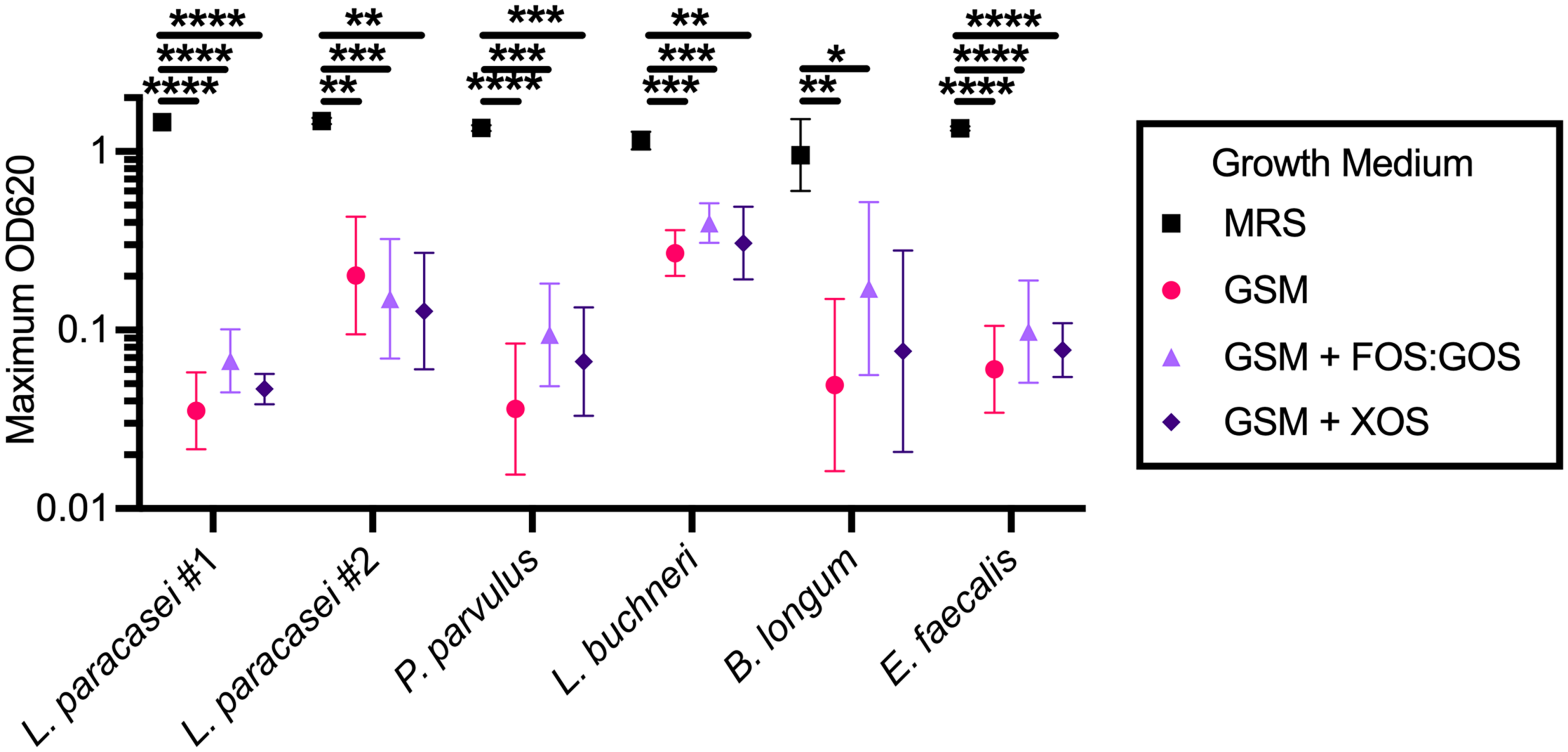
Sauerkraut Lactobacillaceae isolates grow in GSM. Four Lactobacillaceae strains isolated from sauerkraut brine (*Lactobacillus paracasei* #1 and #2, *Pediococcus parvulus*, *Lactobacillus buchneri*) and two fecal strains isolated under Lactobacillaceae selective conditions (*Bifidobacterium longum* and *Enterococcus faecalis*) were grown anaerobically in the indicated media. Data represents geometric mean ± geometric SD. All comparisons between growth media within an isolate with *p*-values <0.05 are reported. *p < 0.05, **p < 0.01, ***p < 0.005, ****p < 0.001.

The inability of fermented food-associated Lactobacillaceae to persist in *in vitro* fecal cultures passaged in GSM could be due to an inability of these microbes to grow in GSM. To test this hypothesis, we selected four of the Lactobacillaceae strains isolated from sauerkraut brine and prebiotic-enriched sauerkraut brine samples described above and tested their ability to grow in GSM and GSM + prebiotics. MRS, a medium that supports robust growth of many Lactobacillaceae, was used as a positive control. We also tested two strains isolated from fecal sample 4 for comparison. We observed that all sauerkraut-associated Lactobacillaceae were able to grow in GSM and GSM + prebiotics, although only one strain of *L. buchneri* reached maximal culture densities within fivefold of cultures grown in MRS (Figure 5). One strain of *L. paracasei* (#2) reached maximal mean culture densities within eightfold of cultures grown in MRS, but the two remaining Lactobacillaceae species (*L. paracasei* #1 and *P. parvulus*) had maximal mean culture densities 40-fold (*L. paracasei*) and 38-fold (*P. parvulus*) lower than cultures grown in MRS. Similarly, fecal isolates obtained maximal mean culture densities ~20-fold (*B. longum*) and 23-fold (*E. faecalis*) lower in GSM compared to MRS. Addition of prebiotics to GSM did not significantly increase the ability of Lactobacillaceae to grow, indicating that other factors in addition to fermentable carbohydrates may limit maximal growth of Lactobacillaceae in GSM.

### *In vitro* culture and addition of prebiotics reduce microbial diversity

**Figure 6:**
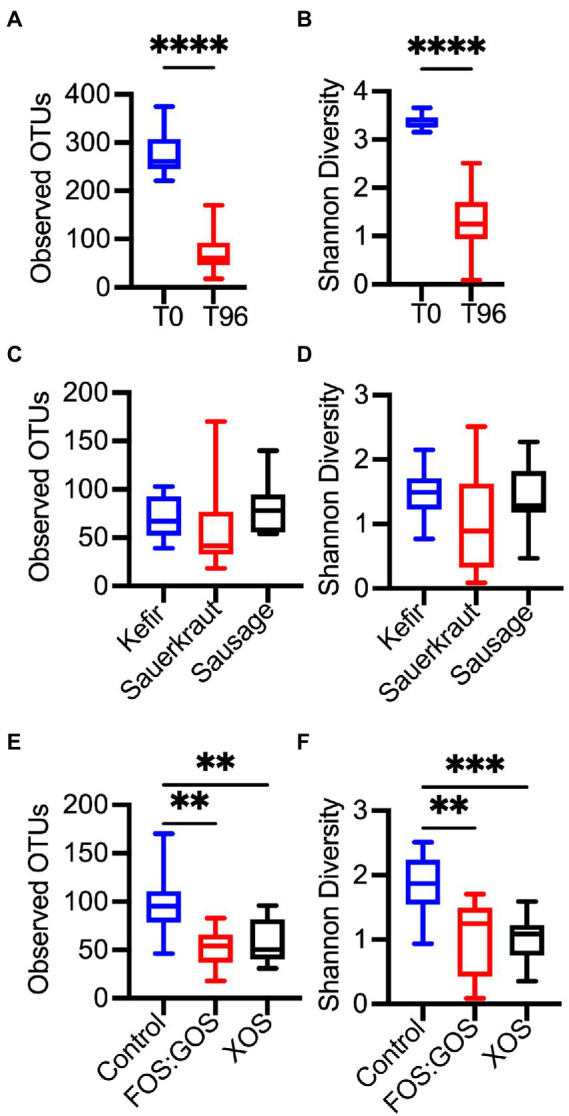
*In vitro* culture and prebiotic treatment alter microbial diversity. Differences in microbial diversity as a result of time in culture **(A,B)**, exposure to a fermented food **(C,D)**, or the presence or absence of prebiotics **(E,F)** was determined for the four fecal cultures described in Figures 1A–C, 2. Microbial richness was assessed through observed OTUs **(A,C,E)** and Shannon Diversity **(B,D,F)**. In **(A,B)**, microbial richness and diversity was compared between replicates collected at the start of *in vitro* culture (T0, *n* = 12) and following 96 h of culture (T96, *n* = 36). In **(C,F)**, microbial richness and diversity were compared between indicated treatments after 96 h of culture (*n* = 12 samples/treatment). Box plots indicate the 25th-75th percentile of values, a horizontal line indicates the median value, and vertical whiskers indicate the minimum and maximum values. All comparisons with *p*-values <0.05 are reported. **p < 0.01, ***p < 0.005, ****p < 0.001.

To determine the overall impact of fermented food-associated microbes and prebiotics on microbiota community structure, 16S rRNA gene sequencing was performed on fermented food samples and samples obtained at time 0 and after 96 h of culturing. Sequences were clustered into operational taxonomic units (OTUs) with ≥99% sequence identity to facilitate strain-level comparisons. Compared to the fecal inocula (T0), all *in vitro* cultures (T96) had reduced levels of richness (Observed OTUs, Figure 6A) and diversity (Shannon Diversity, Figure 6B). This is consistent with our previous study that demonstrated reduced microbial diversity in fecal communities propagated in our culture platform (Kok et al., 2019). The type of fermented food inoculated did not have significant effects on richness (Figure 6C) or diversity (Figure 6D). However, addition of prebiotics led to further decreases in richness (Figure 6E) and diversity (Figure 6F) compared to control cultures passaged *in vitro* for 96 h in the absence of prebiotic.

### Supplementation with prebiotics enhances the persistence of fermented food-associated bacteria

**Figure 7:**
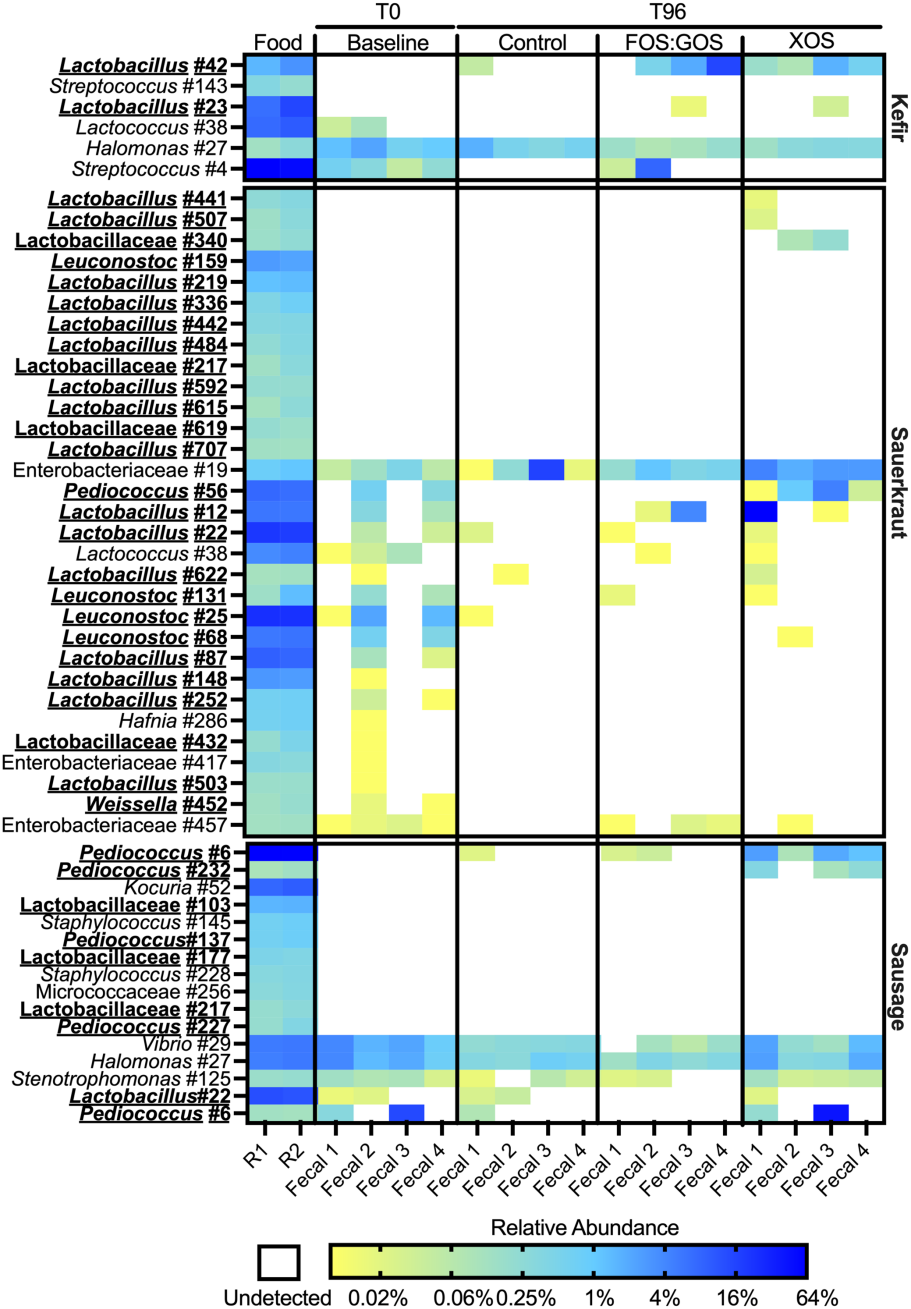
Most fermented food bacteria fail to persist *in vitro*. Persistence of OTUs present at ≥0.1% relative abundance in fermented foods was assessed following culture for 96 h in the presence (FOS:GOS, XOS) or absence (Control) of prebiotics. Relative abundance of OTUs in fecal samples prior to inoculation with fermented foods (Baseline) was also plotted. Shading represents the percent relative abundance. The identity of each OTU is on the left axis; the fermented food inoculated is on the right axis. OTUs that classify as members of the family Lactobacillaceae are indicated by bold-face, underlined typeface. The fermented food replicate number (R1 or R2) and identity of fecal samples are at the bottom of the plot.

To determine how fermented food-associated bacteria persisted *in vitro*, we selected all OTUs detected in both sequenced replicate of a fermented food and compared the abundance of these OTUs across *in vitro* cultures. We also examined abundance of these OTUs in the baseline fecal community (T = 0 sample) to identify those OTUs unique to fermented foods. We identified a total of 29 OTUs for kefir (Supplementary Table 4), 74 OTUs for sauerkraut (Supplementary Table 5) and 54 OTUs for sausage (Supplementary Table 6). 55–80% of OTUs detected were present in low abundance (≤0.1% relative abundance.) Our initial analyses focused on OTUs that were detected in fermented foods with at least 0.1% relative abundance (Figure 7). For kefir, we observed a total of six OTUs at >0.1% relative abundance. Two of these OTUs were members of the Lactobacillaceae family, three OTUs were members of the Streptococcaceae family, and one OTU was a member of the Halomonadaceae family. Three of these OTUs were not detected in baseline fecal samples (*Lactobacillus* #42, *Streptococcus* #143, and *Lactobacillus* #23), while the remaining OTUs were detected in some (*Lactococcus* #38) or all (*Streptococcus* #4 and *Halomonas* #27) baseline fecal samples. Of the five OTUs that were more abundant in fermented foods than in fecal samples (*Lactobacillus* #42, *Streptococcus* #143, *Lactobacillus* #23, *Lactococcus* #38, and *Streptococcus* #4), none consistently persisted in fecal cultures in the absence of prebiotics (*Lactobacillus* #42 was detected in Fecal 1). Consistent with qPCR results, supplementation with XOS supported the persistence of *Lactobacillus* #42 in all fecal cultures, as well as *Lactobacillus* #23 in fecal culture 3. Supplementation with FOS:GOS enhanced the persistence of *Lactobacillus* #42 in 3 of 4 fecal cultures (Fecal 2–4), as well as *Streptococcus* #4 in fecal cultures 1 and 2, and *Lactobacillus* #23 in fecal culture #3. We could not draw conclusions about the persistence of *Halomonas* #27 from kefir, as this OTU was also present at similar levels in fecal cultures at baseline and persisted across all treatments tested. Similar trends were observed for lower abundance OTUs from kefir: 83% of low abundance OTUs (15 OTUs total) were not detected in fecal cultures after 96 h of cultivation in the absence of prebiotics (Supplementary Table 4). For the remaining three low abundance OTUs that were detected in fecal samples, two were *Lactobacillus* OTUs detected at 1 and 3 reads in Fecal culture #4. The third OTU was *Bifidobacterium* #3, which was detected in higher abundance in baseline fecal samples (discussed more below). With the exception of *Bifidobacterium* #3, the addition of prebiotics did not enhance the persistence of these low abundance FAB.

While the sauerkraut microbiota contained a larger number of OTUs than kefir, similar trends were observed regarding the persistence of sauerkraut bacteria over culture time. After filtering out low abundance OTUs (<0.1% relative abundance), 31 OTUs remained. 26 OTUs were members of the Lactobacillaceae, 4 OTUs were members of the Enterobacteriaceae and 1 OTU was a member of the Streptococcaceae family. 13 OTUs were only detected in fermented foods. None of these OTUs persisted in control cultures and only three OTUs (*Lactobacillus* #441, *Lactobacillus* #507, and Lactobacillaceae #340) could be detected following XOS supplementation; none could be detected following FOS:GOS supplementation (Figure 7). There were seven OTUs that were more abundant in sauerkraut than baseline fecal samples whose persistence was enhanced by the addition of XOS (*Pediococcus* #56, *Lactobacillus* #12, *Lactobacillus* #22, *Lactococcus* #38, *Lactobacillus* #622, and *Leuconostoc* #131), although only *Pediococcus* #56 persisted across all fecal cultures. Persistence of three OTUs was enhanced by FOS:GOS (*Lactobacillus* #12, *Lactococcus* #38, and *Leuconostoc* #131) in at least one fecal sample. We could not draw conclusions about the persistence of *Enterobacteriaceae* #19 from sauerkraut, as this OTU was also present at similar levels in fecal cultures at baseline and persisted across all treatments tested. Similar trends were observed for lower abundance OTUs (Supplementary Table 5). Amongst low abundance OTUs, XOS supplementation enhanced low level persistence of *Weissella* #332 and *Acinetobacter* #443.

The sausage microbiota contained 16 OTUs after filtering out those with low abundance. 10 OTUs were only found in sausage; two of these OTUs (*Pediococcus* #6 and *Pediococcus* #232) were able to persist in the presence of XOS supplementation. FOS:GOS supplementation also enhanced persistence of *Pediococcus* #6. Persistence of three OTUs (*Vibrio* #29, *Halomonas* #27, and *Stenotrophomonas* #125) present at similar levels across fermented food and baseline samples could not be determined. Similarly, *Lactobacillus* #22 and *Bifidobacterium* #9 were detected in both fermented foods and a subset of fecal samples and so we cannot determine conclusively whether OTUs that persisted originated from the fecal samples or the sausage.

### Prebiotics enhance persistence of *Lactobacillus* and *Bifidobacterium*

**Figure 8:**
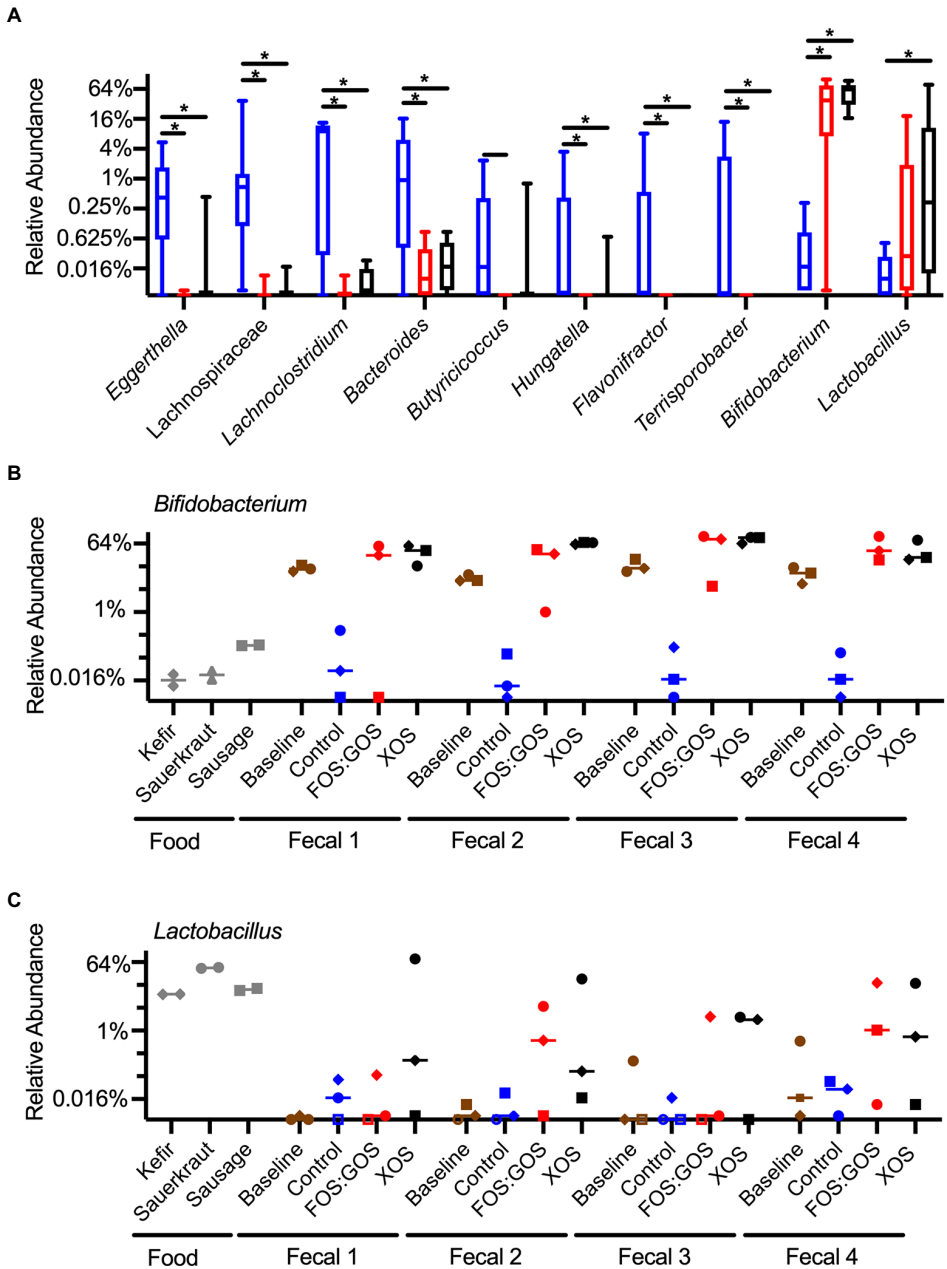
Prebiotic addition favors the enrichment of *Bifidobacterium* and *Lactobacillus* species. **(A)** Genera that differ significantly in abundance between control, FOS:GOS, and XOS-treated cultures after 96 h of *in vitro* culture. Boxes indicate the 25th–75th percentile, horizontal line indicates the median, and vertical whiskers indicate minimum and maximum values. All statistically significant comparisons are indicated by asterisks. **(B)** The relative abundance of *Bididobacterium* across each replicate sample is plotted with a horizontal line at the median value. **(C)** The relative abundance of *Lactobacillus* across each replicate sample is plotted with a horizontal line at the median value. In **(B,C)**, shapes of symbols indicated the fermented food added to each *in vitro* culture (kefir, diamonds; sauerkraut, circles; sausage, squares). In **(A–C)**, the baseline is set at the limits of detection. In **(C)**, open symbols indicate samples with sequences below the limit of detection, set at baseline for comparison.

To determine how treatment with prebiotics affected specific taxa within *in vitro* cultured communities, we compared differences in relative abundance between genera using ANCOM-BC, a tool for analyzing microbiome composition with bias correction (Lin and Peddada, 2020). We performed analysis at the genus level to minimize potential effects of strain-level differences between fecal communities. When we focused on taxa that were present above 0.1% relative abundance in at least 25% of samples from one or more treatment groups, we found that prebiotic treatment consistently enhanced the growth of *Bifidobacterium* and *Lactobacillus* and suppressed the growth of *Eggerthella,* *Lachnospiraceae*, *Lachnoclostridium, Bacteroides, Butyricoccus, Hungatella, Flavonifractor,* and *Terrisporobacter* (Figure 8A). Treatment with XOS more consistently enhanced the growth of *Bifidobacterium* compared to treatment with FOS:GOS. Although additional taxa were identified that had statistically significant differences between treatments, these taxa were more variable between fecal communities and replicates and did not follow consistent trends (Supplementary Table 7).

To gain a better understanding of the whether the *Bifidobacterium* and *Lactobacillus* species more likely originated from fermented foods or fecal samples, we plotted the abundance of these genera across all samples (Figures 8B,C). From these data, we observed that *Bifidobacterium* species were low in fermented foods (~0.02–0.2% relative abundance) and high in baseline fecal samples (~5–20% relative abundance, Figure 8B). *In vitro* cultivation for 96 h in the absence of prebiotics led to low level persistence of *Bifidobacterium* (~0.01–0.3% relative abundance), but the presence of XOS led to high levels of *Bifidobacterium* (~20–90% relative abundance, Figure 8B). FOS:GOS led to high levels of *Bifidobacterium* in many samples, but results were more variable (Figure 8B). In contrast, *Lactobacillus* levels were high in fermented foods (~10–40%, Figure 8C), low in baseline fecal samples, and higher in some cultures that included prebiotics.

## Discussion

Consumption of fermented foods containing live microorganisms contributes to what has been called the “transient microbiome” (Derrien and van Hylckama Vlieg, 2015). Although the food matrix and specific food constituents may promote survival of these microbes during transit, acids, enzymes, and other host barriers may impair their ability to reach the colon (Guerra et al., 2012; Zhang et al., 2016). Even if microbes from fermented foods successfully survive the stressful conditions of GI transit, the well-adapted autochthonous microbes will likely out-compete allochthonous microbes for nutrients, space, and resources. Thus, fermented food-associated microbes rarely persist and are usually displaced relatively quickly (Pasolli et al., 2020).

In this study, we used an *in vitro* fecal cultivation model to assess persistence of fermented food-associated bacteria and the effect of prebiotics on persistence. The experimental design was intended to approximate normal colonic transit rates of 12–24 h (Guerra et al., 2012), achieved using stepwise dilutions. We observed that fermented food microbes failed to persist during these *in vitro* cultures for all four of the donor samples tested. After 4 days in culture, there were no detectable Lactobacillaceae and little to no FAB sequences for any of the fecal communities with any of the fermented foods.

Supplementation with prebiotics enhanced persistence of fermented food-associated Lactobacillaceae and a small number of other species (*Streptococcus* from kefir; *Acinetobacter* from sauerkraut). However, persistence was both prebiotic- and subject-dependent. Thus, when sauerkraut microbes were supplemented with XOS, Lactobacillaceae persisted at nearly the original inoculation level (about 10^6^–10^7^ CFU/mL) for three of four donor samples (Fecal 1, 2, and 4). In contrast, persistence was observed for only one of the four samples supplemented with FOS:GOS (Fecal 2). Based on 16S rRNA gene sequencing, this observation may be due to the ability of XOS to select for persistence of a strain of *Pediococcus* in 3 of 4 samples (Fecal 2–4) and *Lactobacillus* in the other sample (Fecal 1). A similar outcome was observed for Lactobacillaceae from sausage, where XOS enhanced persistence of Lactobacillaceae in all four subjects, but persistence was subject-dependent for the GOS:FOS treatment (Lactobacillaceae persisted in Fecal 1 and 4). Again, 16S rRNA gene sequencing indicated this may be due to the ability of XOS to select for the persistence of strains of *Pediococcus*. Both prebiotics supported the persistence of kefir-associated Lactobacillaceae across all fecal samples. 16S rRNA gene sequencing indicated that may be due to the persistence of a single *Lactobacillus* strain (designated as *Lactobacillus* OTU #42), although this was not observed for fecal sample 1 grown in the presence of FOS:GOS. In this sample, 16S rRNA gene sequences were instead detected from 24 different OTUs that were members of the Lactobacillaceae family (Supplementary Table 3). Thus, for this small set of fecal communities and fermented foods, XOS consistently contributed to greater persistence of fermented food-associated Lactobacillaceae than a FOS:GOS mixture. Some of this greater efficacy of XOS may be attributed to its potential ability to support persistence of *Pediococcus* strains present in sauerkraut and sausage. Future studies with larger numbers of fecal samples and fermented foods with different FAB composition could provide more insights into these differential responses, which were limited by the small sample size of this study.

In addition to our observation that prebiotics enhanced the persistence of a subset of FAB, we also observed that prebiotic supplementation enhanced growth of *Bifidobacterium*. While *Bifidobacterium* sequences were detected at very low levels in fermented foods, reads were much higher in baseline fecal samples, suggesting that *Bifidobacterium* species whose growth was enhanced by prebiotic treatment were most likely of fecal origin. Future studies using whole genome shotgun metagenomics to provide greater strain-level resolution would be needed to definitively answer this question.

While prebiotic treatment enhanced the growth of *Lactobacillus* and *Bifidobacterium*, prebiotic treatment also led to reduced levels of several genera. These reductions in genera are consistent with the alpha diversity measures reported for control and prebiotic-treated cultures, which showed lower levels of observed OTUs in prebiotic-treated cultures. While higher microbial diversity is often associated with a healthy status, predominance of organisms with potential probiotic capabilities selected by prebiotic treatment may mitigate the potential negative effects of reduced diversity.

Overall, these results are consistent with human clinical data (summarized in Derrien and van Hylckama Vlieg, 2015). Specifically, fermented food-associated microbes do not persist in the gastrointestinal environment, whether *in vivo* or *in vitro*. Thus, any microbiota-mediated health benefits these foods may provide to the host would likely be conferred only if those foods were consumed regularly. This data are consistent with the work by Pasolli et al. (2020), who analyzed human clinical samples and metagenome-assembled genomes and demonstrated that food, and fermented foods in particular, were the major source of lactic acid bacteria in the human gut. These authors noted that although prevalence of LAB varied, most were present at low abundance. Among the exceptions were *Streptococcus thermophilus* (0.6% relative abundance) and *Lactococcus lactis* (0.4% relative abundance) that were likely consumed regularly through consumption of yogurt and cheese.

Despite the transient nature of fermented food-associated bacteria observed *in vivo* and in our *in vitro* cultures, our data contributes to the emerging evidence that persistence of FAB could be enhanced by consumption of prebiotics or other microbiota-accessible carbohydrates. According to a recent metagenome analysis of fermented foods, the primary driver of microbiota composition is substrate availability (Leech et al., 2020). Most fermented foods made using LAB contain readily fermentable sugars that exist naturally, are enzymatically formed from larger molecules, or are intentionally added (Marco et al., 2021). However, in the colon, these substrates are usually absent, which explains, in part, why these bacteria do not persist well in the colonic environment. Thus, providing suitable resources in the form of prebiotic substrates or microbiota-accessible carbohydrates may offer a mechanism to enhance persistence and abundance of these bacteria in the gut.

The potential health benefits of fermented foods, including FAB, are now the subject of considerable research attention. Fermented foods have long served as good sources of proteins, mineral, vitamins, and other nutrients, but it is the live microbes that are now of interest due to their suggested role in enhancing gut health (Marco et al., 2017). Based on the results presented here and in the other studies described above, such benefits would require regular consumption of these foods due to the transient nature of their microbiomes and may be enhanced by consumption of prebiotics or other microbiota accessible carbohydrates that selectively enhance the growth of FAB.

## Data availability statement

The data presented in this study are deposited in the NCBI short read archive repository, accession number PRJNA824793.

## Ethics statement

The studies involving human participants were reviewed and approved by University of Nebraska Institutional Review Board. The patients/participants provided their written informed consent to participate in this study.

## Author contributions

RH and CC designed the study. CC collected data. CK, JMA, RH, and CC analyzed data and interpreted results, prepared the manuscript, and approved the final version of the manuscript. All authors contributed to the article and approved the submitted version.

## Funding

The project was supported by funding from the University of Nebraska–Lincoln UCARE program. CC was also supported by a graduate fellowship provided by the Department of Food Science and Technology, University of Nebraska-Lincoln. CK was supported by a fellowship provided by the Nebraska Food for Health Center. Computational analysis was completed utilizing the Holland Computing Center of the University of Nebraska, which received support from the Nebraska Research Initiative.

## Conflict of interest

RH is a co-founder of Synbiotic Health, a manufacturer of probiotic microbes. JMA has a financial interest in Synbiotic Health.

The remaining authors declare that the research was conducted in the absence of any commercial or financial relationships that could be construed as a potential conflict of interest.

## Publisher’s note

All claims expressed in this article are solely those of the authors and do not necessarily represent those of their affiliated organizations, or those of the publisher, the editors and the reviewers. Any product that may be evaluated in this article, or claim that may be made by its manufacturer, is not guaranteed or endorsed by the publisher.

## Footnotes

^1^ https://atima.research.bcm.edu
