# Supplementary material for: Prebiotics enhance persistence of fermented-food associated bacteria in in vitro cultivated fecal microbial communities
Source: Front Microbiol. 2022 Sep 2;13:908506. doi: 10.3389/fmicb.2022.908506 (PMC9479011; doi:10.3389/fmicb.2022.908506)
Supplement: Supplementary file 2 [file Image_1.pdf]

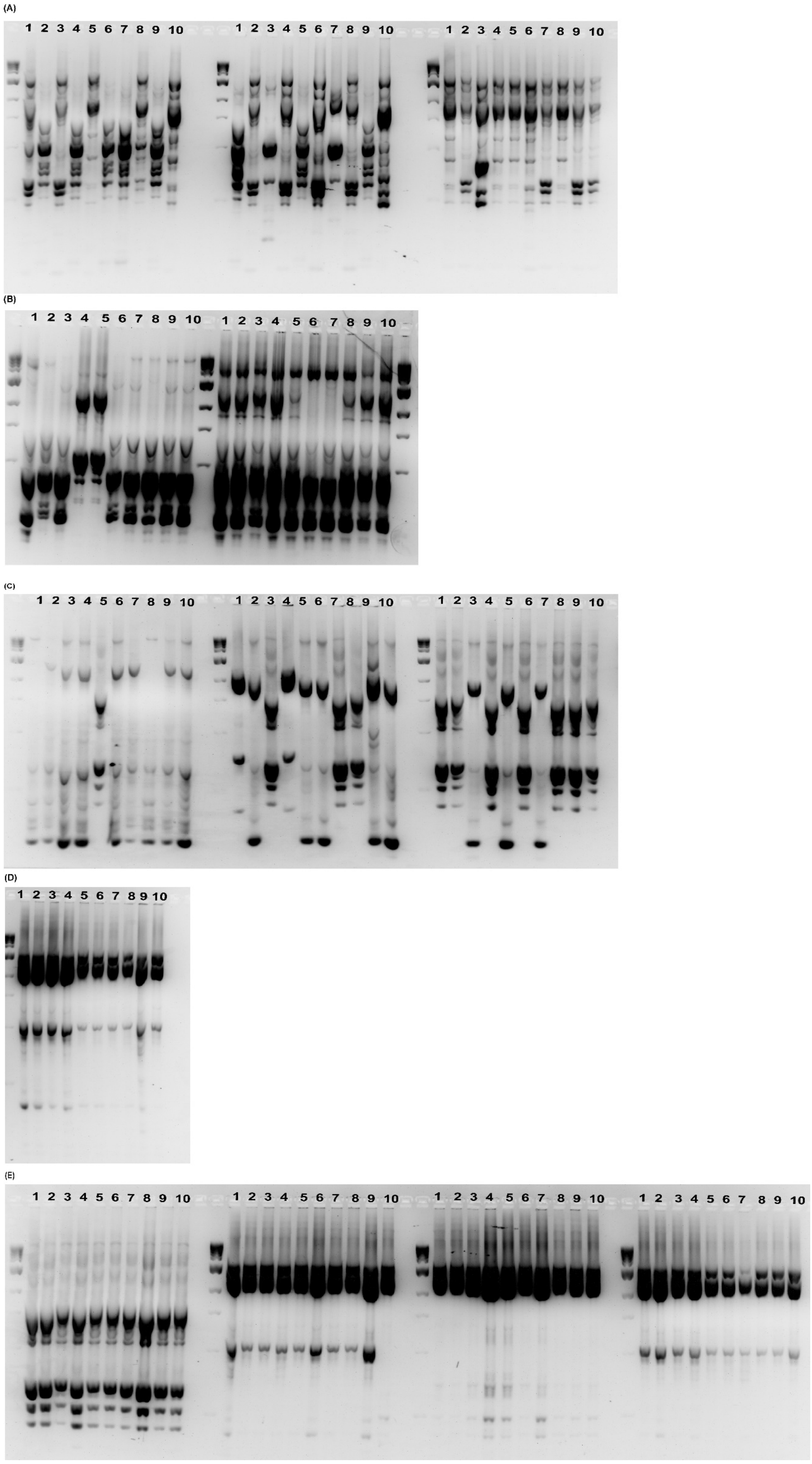
(A)
(B)
(C)
(D)
(E)

Supplementary Figure 1. Full gel images of RAPD fingerprinting of isolates from sauerkraut brine, prebiotic-enriched sauerkraut brine, and prebiotic-enriched *in vitro* fecal cultures (**A**) RAPD gel images of isolates from sauerkraut brine (left, 1-10), sauerkraut brine selectively enriched with 1% XOS (middle, 1-10), and sauerkraut brine selectively enriched with 0.5% FOS and 0.5% GOS (right, 1-10). Unique RAPD profiles from sauerkraut were observed in Lanes 1, 3, 4, 8, and 10 and were shown in Figure 4A Lanes 1-5, respectively. Unique RAPD profiles from sauerkraut enriched with 1% XOS were observed in Lanes 1, 2, 3, and 10 and were shown in Figure 4A Lanes 6-9, respectively. Unique RAPD profiles from sauerkraut enriched with 0.5% FOS and 0.5% GOS were observed in Lanes 1, 2, and 3, and were shown in Figure 4A Lanes 10-12, respectively. (**B**, **C**) RAPD gel images of isolates from *in vitro* fecal cultures supplemented with 1% XOS from Fecal 1 (**B**; left, 1-10), Fecal 2 (**B**; right, 1-10), Fecal 3 (**C**; left, 1-10), or Fecal 4 (**C**; middle, 1-10). Unique RAPD profiles from Fecal 1 were observed in Lanes 5 and 6 and were shown in Figure 4B Lanes 1-2, respectively. Unique RAPD profiles from Fecal 2 were observed in Lanes 6 and 8 and were shown in Figure 4B Lanes 3-4, respectively. Unique RAPD profiles from Fecal 3 were observed in Lanes 1, 3, and 5 and were shown in Figure 4B Lanes 5-7, respectively. Unique RAPD profiles from Fecal 4 were observed in Lanes 1, 2 and 3 and were shown in Figure 4B Lanes 8-10, respectively. (**D**, **E**) RAPD gel images of *in vitro* fecal cultures supplemented with 0.5% FOS and 0.5% GOS from Fecal 1 (**D**, 1-10), Fecal 2 (**E**; left, 1-10), Fecal 3 (**E**; middle left, 1-10), and Fecal 4 (**E**; middle right, 1-10). Unique RAPD profiles were observed in Lanes 4 and 5 from Fecal 1 and were shown in Figure 4C Lanes 1-2, respectively. Unique RAPD profiles were observed in Lane 1 from Fecal 2 and were shown in Figure 4C Lane 3. Unique RAPD profiles were observed in Lane 1 from Fecal 3 and were shown in Figure 4C Lane 4. Unique RAPD profiles were observed in Lane 1 from Fecal 4 and were shown in Figure 4C Lane 5. To show full gel images, we have included two panels (**C**; right, 1-10) and (**E**; right, 1-10) that were not a part of this study. Isolates that we identified as unique RAPD profiles based upon visual observation of differences in fingerprinting were further characterized by 16S rRNA gene sequencing, with at least one isolate chosen from each fecal sample, sauerkraut brine sample, and prebiotic-enriched sauerkraut brine samples.
